# Black Women’s Confidence in the Genetic Information Nondiscrimination Act

**DOI:** 10.3390/ijerph16245112

**Published:** 2019-12-14

**Authors:** Arnethea L. Sutton, Alesha Henderson, Alejandra Hurtado-de-Mendoza, Erin Tanner, Mishaal Khan, John Quillin, Vanessa B. Sheppard

**Affiliations:** 1 Department of Health Behavior and Policy, Virginia Commonwealth University School of Medicine, Richmond, VA 23298, USA; hendersonan2@vcu.edu (A.H.); khanm6@vcu.edu (M.K.); vanessa.sheppard@vcuhealth.org (V.B.S.); 2 Department of Oncology, Georgetown University Medical Center, Washington, DC 20007, USA; ahd28@georgetown.edu; 3 Greater Washington Maternal-Fetal Medicine and Genetics, Rockville, MD 47872, USA; tannere@mymail.vcu.edu; 4 Department of Human and Molecular Genetics, Virginia Commonwealth University School of Medicine, Richmond, VA 23298, USA; john.quillin@vcuhealth.org; 5 Office of Health Equity and Disparities Research, VCU Massey Cancer Center, Richmond, VA 23298, USA

**Keywords:** genetic counseling and testing, Genetic Information Nondiscrimination Act, hereditary breast and ovarian cancer, black women

## Abstract

Black women at-risk for hereditary breast and ovarian cancer (HBOC) continue to underutilize genetic counseling and testing (GCT). One reason for this disparity is a fear of discrimination from insurance companies if identified as high-risk. The Genetic Information Nondiscrimination Act (GINA) was enacted to protect against this type of discrimination; however, Black women’s levels of confidence in this law are unknown. In this descriptive study, we sought to (1) assess Black women’s confidence in the GINA law and (2) identify multilevel factors related to their confidence in GINA. Ninety-four Black women at-risk of HBOC completed surveys that assessed intrapersonal, interpersonal, and structural factors. Multiple regression analysis determined factors associated with confidence in GINA. Most women were ≤50 years of age (66.0%) and about half never had a cancer diagnosis (51.1%). Confidence in GINA was moderate (mean = 10.67; standard deviation = 2.54; range = 5–15). Women who valued GCT reported more confidence in GINA (β = 0.345; CI 0.017 to 0.673; *p* = 0.040). Lack of confidence in GINA may serve as a barrier to seeking GCT. Efforts to increase the perceived value of GCT among Black women could be benefited by increasing awareness of national efforts towards privacy protections of genetic information.

## 1. Introduction

Breast cancer is the most common cancer among women in the United States and racial disparities exist with regard to cancer outcomes. Black women have the highest mortality rate at 29.5 per 100,000 women, compared to the mortality rate of non-Hispanic white women at 20.8 per 100,000 women [1]. Black women also have a higher incidence of triple negative breast cancer (TNBC), a more aggressive form of breast cancer that is very difficult to treat [2,3,4].

Genetic counseling and testing (GCT), specifically for mutations in *BRCA1/2* genes for breast and ovarian cancers, is a beneficial service that assesses the risk of developing hereditary cancers. Even with higher incidence of TNBC and higher mortality rates due to breast cancer, Black women are less likely to seek genetic testing (GCT) than their White counterparts [5,6,7,8]. Even Black women who are considered high risk (e.g., first-degree relative with breast cancer) for hereditary breast cancer have been shown to underutilize *BRCA1/2* testing [6]. Various reasons contribute to Black women’s underutilization of GCT, including lack of knowledge, lack of physician recommendation, high cost of GCT, limited access to health care, and medical distrust [7,9,10,11]. Another major concern is a fear of discrimination due to disclosure of testing results [12,13].

On 21 May 2008, Congress enacted the Genetic Information Nondiscrimination Act (GINA) to prevent misuse of genetic information. This act banned health insurance companies as well as employers from discriminating based on genetic information obtained from genetic tests [14]. However, there are limitations to GINA. This act does not cover life insurance policies, meaning individuals who are asymptomatic for diseases can be denied life insurance policies based on this information [15]. Also, this act only protects individuals from discrimination up until the point of the disease manifestation [14]. Genetic counselors reported that only about 15% of patients were aware of GINA prior to the discussion with a genetic counselor [16]. In a larger online survey, Green and colleagues found that 79% of respondents were not aware of GINA and more strikingly, once informed, 30% reported a greater fear of discrimination [17]. Since GCT is becoming more accessible to individuals, there is a greater need to understand the confidence individuals have in the protection of their genetic information. One cohort study that assessed at-risk women’s attitudes about GINA reported that women who had a prior knowledge of GINA had more concerns with insurance discrimination than women who had no prior knowledge of GINA. Findings for this study were not generalizable to Black at-risk women as they only comprised 2.1% of the sample [18]. Further, as Black women express experiences and concerns with discrimination and mistrust in healthcare and the government [11], there is a need to elucidate factors related to their confidence in GINA.

The social ecological model was employed as a guiding conceptual framework to examine the relationships of intrapersonal (e.g., demographics), interpersonal (e.g., race-based healthcare discrimination), and structural factors (e.g., medical mistrust), on high-risk Black women’s confidence in GINA. Other studies found that multilevel factors predict individuals’ attitudes or feelings about laws; therefore, the social ecological model was appropriate given its employment of multilevel factors [19,20]. The aims of this study were to (1) assess Black women’s confidence in GINA and (2) examine the relationship between GINA and interpersonal, intrapersonal, and structural factors.

## 2. Methods

### 2.1. Sample and Setting

This is a secondary analysis of a cross-sectional study that included women who were at-risk for hereditary breast and ovarian cancer. As such, in order to be eligible for the study, women had to respond "yes" to at least one of the following criteria: (1) healthy woman with ≥1 first-degree relative with breast and/or ovarian cancer; (2) woman diagnosed with breast cancer at age ≤50; or (3) woman diagnosed with breast and/or ovarian cancer at >50 with either one first-degree relative or two second-degree relatives with breast and/or ovarian cancer. Women also had to be at least 21 years of age and able to read and understand English.

Using a convenience sampling method, participants (*n* = 100) were recruited from clinical and community settings including a professional women’s basketball game, a waiting area of a breast care clinic, a faith-based community event, and a grocery store in Washington, DC. Twenty-one women were recruited from the clinic setting while the rest were recruited via community outreach efforts. Women who met inclusion criteria for being high-risk for *BRCA1/2* mutations were contacted for a semistructured phone interview that lasted approximately 30 min. Women received a $25 gift certificate for their participation. Details of the study procedure may be found elsewhere [7]. Informed consent was obtained from all women. All procedures were approved by the institutional review boards for the parent study and for this secondary analysis.

### 2.2. Measures

GINA Confidence. The GINA confidence scale includes three five-point Likert items related to confidence in the protections afforded by GINA. Prior to asking participants to respond to these items, the clinical research assistant gave a brief description of the law to acclimate those who may have been unaware. After a description of the law, participants respond to items: (1) having this law in place is important in my decision to test for *BRCA* mutations: (2) I am confident the GINA Law will prevent discrimination based on genetic health information; and (3) because of this law, I am confident that if I were to have a genetic test, the results would be protected [7]. Responses ranged from “strongly disagree” to “strongly agree” (score range = 3–15) with higher scores indicating greater confidence in GINA (Cronbach’s alpha = 0.740).

Intrapersonal Factors: Demographic factors evaluated included age, marital status, education, and employment status. Participants’ attitudes towards GCT were assessed using a 15-item four-point Likert scale with responses ranging from “very important” to “not at all important" (Cronbach’s alpha = 0.827) [18]. Scores ranged from 15–60 with higher scores indicating more favorable attitudes. Knowledge of breast cancer genetics was assessed using 13 true or false items relating to the hereditary breast cancer mutations, inheritance, and cancer risks (Cronbach’s alpha = 0.648) [19]. Higher scores indicated higher breast cancer genetics knowledge (score range = 0–13). Perceived behavioral control was assessed using three items related to control, choice, and free-decision making regarding GCT (e.g., it is my choice whether or not I receive genetic counseling and testing). Individuals were asked to answer items using a four-point Likert scale ranging from “completely disagree” to “completely agree” (score range = 3–12) (Cronbach’s alpha = 0.861) [20]. Higher scores corresponded to a greater perceived control in undertaking GCT. Value of genetic counseling and testing was assessed using a three-item five-point Likert scale relating to importance of genetic counseling and testing and genetic testing risk (score range = 3–15) (Cronbach’s alpha = 0.590). Higher scores corresponded with greater value in GCT. Confidence in GCT was assessed using five four-point Likert scale items relating to confidence in coping with results, ability to make decisions regarding risk-reducing surgeries or screening, and ability to communicate with family members. Responses ranged from “not at all confident” to “very confident” (score range = 4–16) (Cronbach’s alpha = 0.733) [21].

Interpersonal Factors: Bird and Bogart’s (2001) [22] race-based experiences scale was used to assess participants’ experiences with discrimination during their healthcare interactions. This measure consisted of seven yes/no items (score range = 0–7) (Cronbach’s alpha = 0.873). Higher scores indicated more experiences of healthcare discrimination.

Structural Factors: Medical mistrust was assessed using seven five-point Likert scale items relating to perceptions of and trust in healthcare providers or healthcare organizations. Responses ranged from “strongly disagree” to “strongly agree” (score range = 7–30) (Cronbach’s alpha = 0.76) [23]. A higher score corresponded with higher medical mistrust. Perceived difficulty of receiving genetic testing was assessed using six five-point Likert scale items asking women about barriers to obtaining genetic testing (score range = 6–30) (Cronbach’s alpha = 0.779). A higher score indicated higher perceived difficulty.

### 2.3. Data Analysis

This analysis included 94 women, as some women were excluded due to missing data. The demographic and frequency table was summarized. Analysis of variance (ANOVA) was performed to assess relationships between each continuous independent variable and confidence in GINA. Chi-square was used to assess relationships between categorical variables and confidence in GINA. All variables shown in Table 1 were included in the model. The variables were selected through a stepwise procedure in the multivariable regression model. All statistical analysis was conducted with IBM SPSS 25.

## 3. Results

A total of 94 Black women were included in these analyses. The mean age of women was 44.9 years. Most women were 50 years of age or older (66.0%), were married (41.5%), had greater than a high school education (83.0%), and were employed full time (75.5%). About half of women were not diagnosed with breast or ovarian cancer (51.1%) (Table 1).

**Figure 1:**
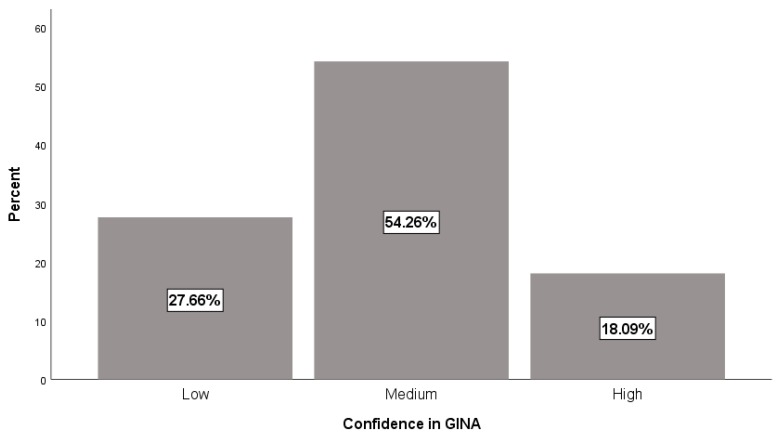
Levels of women’s confidence in GINA.

The total range of scores for women’s confidence in GINA was 5–15 with the mean score of 10.67 (standard deviation = 2.54). In Figure 1, we illustrate women’s confidence levels in GINA (categorized based on tertiles). A majority of women had "low to medium" confidence in GINA (81.9%) (Figure 1). In bivariate analyses, education level (*p* = 0.04), perceived behavioral control (*p* = 0.001), and perceived race-based discrimination (*p* = 0.021) were the only factors significantly related to confidence in GINA. The model explained 25% of the variability in confidence in GINA. Greater confidence in GINA was observed among women who reported greater value in GCT (β = 0.345; CI 0.017 to 0.673; *p* = 0.040).

## 4. Discussions

To our knowledge, this is the first study to assess confidence in GINA in Black high-risk individuals. Overall, high-risk Black women in our sample were confident in GINA. This is of particular importance as studies indicate that most individuals are unfamiliar with GINA [24,25,26]. We assessed three categories of factors; however, intrapersonal was the only category with significant factors related to our outcome. Higher confidence in GINA was significantly related to the degree to which women value GCT.

Greater confidence in GINA was reported by women who valued GCT. Given the type of questions women were asked to assess their value of GCT (e.g., the risk of discrimination from a positive genetic test result is not worth it), it is possible that women who expressed lower value in GCT have heightened concerns about discrimination which limits their ability to fully trust GINA or, while at-risk, they do not feel that GCT warrants the risk of a breach of their personal information [18]. Women who value testing may do so because they may feel a sense of reassurance and may understand the benefits with regard to knowing future risk [6]. Developing interventions that seek to emphasize the importance and value of GCT to Black women at-risk of *BRCA1/2* mutations are needed. Primary care and women’s health providers may be key to initiating important conversations for at-risk women.

In bivariate analysis, women with at least some college education reported greater confidence in GINA than women with a high school education or less. Chapman and colleagues [27] reported a positive correlation between education and knowledge of genetic concepts. Although we did not assess knowledge of GINA, this finding may also serve as a proxy for women’s understanding of the law and how GINA may protect them from genetic discrimination. Therefore, women with more education may have understood the implications of the law more so than their counterparts. This finding may highlight the need to examine how information about GINA is presented. This may include use of low literacy material. Providers should foster an atmosphere that is conducive to two-way dialogue inclusive of opportunities to ask clarifying questions [18]. It may be helpful to encourage women to bring a family member or friend along, as this person may help facilitate conversations.

In our study, greater perceived behavioral control in obtaining GCT was associated with greater confidence in GINA. This is an interesting finding, as it may suggest that women who do not feel that they have complete control over a decision to undergo GCT may also lack confidence in the government to provide protection against discrimination related to GCT. To our knowledge, this finding has not been reported elsewhere. Studies indicate the improvement of one’s perceived behavioral control after receiving GCT [28,29], but there is a clear need to intervene in women prior to receiving GCT if there is to be any improvement in GCT uptake in at-risk Black women. Given the need for at-risk women to consider GCT, future research must identify factors related to women’s lack of perceived behavioral control in obtaining GCT.

Although we report valuable findings, there are limitations to note. Our sample size was small. Since racial disparities exist with regard to GCT uptake, future work should focus on larger numbers of Black women. Findings from our study are not generalizable to non-Black women or women who do not reside in urban areas. Also, women’s awareness or knowledge of GINA was not assessed. Cronbach’s alphas for some scales, particularly value of genetic counseling, were not ideal. This may be due to our small sample size and the low number of items in those scales. Lastly, we limited our sample to women at-risk for hereditary breast and ovarian cancer; therefore, findings are not generalizable to individuals at-risk of other cancers and genetic mutations.

## 5. Conclusions

This study demonstrates a need to identify strategies to educate women on GINA in an effort to build confidence in the law. Not only is this salient to potentially improving rates of GCT uptake, but also the implications for how this law may impact engagement in clinical trials and other studies involving biospecimen provision and genetic testing are profound [30], particularly as there is an underrepresentation of Black individuals in research and clinical trials. Future studies are needed to determine how GINA may impact women’s decisions to receive GCT. Although implemented 10 years ago, changes to the law may be inevitable as courts disagree over the meaning of the term “genetic information” [31]. If changes occur, it will be pertinent to educate at-risk women of changes by tailoring information to the population with the goal of bolstering confidence in GINA and improving GCT rates in Black women.

## Figures and Tables

**Table 1:** Intrapersonal, interpersonal, and structural factors and confidence in the Genetic Information Nondiscrimination Act (GINA) (*n* = 94).

Variables	*n* (%)	Mean (SD)	*p*-Value
**Intrapersonal**			
Age (M ± SD)	44.9 (11.4)		0.20
≤50 years	62 (66.0)	10.8 (2.5)	
>50 years	32 (34.0)	10.4 (2.7)	0.44
Marital Status			0.36
Married/Living as married	39 (41.5)	10.6 (2.7)	
Single (never married)	38 (40.4)	10.8 (2.7)	
Other	17 (18.1)	10.7 (2.9)	
Education Level Attained			0.04 *
Less than or equal to high school	16 (17.0)	11.6 (2.3)	
Greater than high school	78 (83.0)	10.5 (2.6)	
Insurance Status			0.13
Has insurance	87 (92.6)	10.7 (2.6)	
Does not have insurance	7 (7.4)	10.7 (1.7)	
Work Arrangement			0.84
Full time employed	71 (75.5)	10.9 (2.5)	
Not full time employed	23 (24.5)	9.9 (2.6)	
Cancer Status			0.24
Diagnosed	46 (48.9)	10.9 (2.6)	
Not diagnosed	48 (51.1)	10.5 (2.5)	
GCT Engagement			0.70
Yes	15 (22.7)	10.6 (2.9)	
No	51 (77.3)	10.8 (2.7)	
Attitude toward GCT		41.0 (4.0)	0.63
Perceived Behavioral Control		14.0 (2.0)	0.001 ^‡^
Knowledge of GCT		8.3 (1.9)	0.64
Value in GCT		11.3 (1.9)	0.06
Confidence in GCT		13.8 (2.2)	0.12
**Interpersonal**			
Race-Based Discrimination		2.4 (2.4)	0.021 ^*^
**Structural**			
Medical Mistrust		24.9 (3.9)	0.36
Difficulty obtaining GCT		13.0 (3.6)	0.42

* *p* < 0.05; ^‡^ *p* < 0.01.
